# Behavioural and Physiological Effects of Finely Balanced Decision-Making in Chickens

**DOI:** 10.1371/journal.pone.0108809

**Published:** 2014-10-02

**Authors:** Anna C. Davies, Christine J. Nicol, Mia E. Persson, Andrew N. Radford

**Affiliations:** 1 School of Clinical Veterinary Science, University of Bristol, Bristol, United Kingdom; 2 Avian Behavioural Genomics and Physiology Group, Linköping University, Linköping, Sweden; 3 School of Biological Sciences, University of Bristol, Bristol, United Kingdom; CNR, Italy

## Abstract

In humans, more difficult decisions result in behavioural and physiological changes suggestive of increased arousal, but little is known about the effect of decision difficulty in other species. A difficult decision can have a number of characteristics; we aimed to monitor how finely balanced decisions, compared to unbalanced ones, affected the behaviour and physiology of chickens. An unbalanced decision was one in which the two options were of unequal net value (1 (Q1) vs. 6 (Q6) pieces of sweetcorn with no cost associated with either option); a finely balanced decision was one in which the options were of equal net value (i.e. hens were "indifferent" to both options). To identify hens' indifference, a titration procedure was used in which a cost (electromagnetic weight on an access door) was applied to the Q6 option, to find the individual point at which hens chose this option approximately equally to Q1 via a non-weighted door. We then compared behavioural and physiological indicators of arousal (head movements, latency to choose, heart-rate variability and surface body temperature) when chickens made decisions that were unbalanced or finely balanced. Significant physiological (heart-rate variability) and behavioural (latency to pen) differences were found between the finely balanced and balanced conditions, but these were likely to be artefacts of the greater time and effort required to push through the weighted doors. No other behavioural and physiological measures were significantly different between the decision categories. We suggest that more information is needed on when best to monitor likely changes in arousal during decision-making and that future studies should consider decisions defined as difficult in other ways.

## Introduction

Humans and non-human animals are faced with decisions in all aspects of their lives [1], [2]. The process of decision-making in humans is associated with changes in behaviour and physiology, including galvanic skin responses and heart-rate (HR) [3], indicative of increased arousal. Moreover, more difficult decisions lead to increases in such response parameters e.g. [4]–[7]. Although behavioural and physiological processes have been studied during decision-making in chickens [8], little is known about whether decisions of greater difficulty result in increased arousal in non-human species. Procedures involving decision-making are used widely in animal research, such as when testing preferences for environmental or social resources [9]–[11]. For example, choice tests have been used to determine chickens' preferred dustbathing substrate [12] and lighting [13]. Since the results of these tests can have practical, political and welfare implications, any procedural influences must be considered as part of an overall interpretation [14], [15].

A complicating factor is that decisions may be “difficult” in more than one way. A difficult decision might, for instance, involve finely balanced options (e.g. two options with the same net value: [16]), have the risk of a critical outcome (e.g. a risk of predation: [17]), have an ambiguous outcome (e.g. insufficient information available: [18]), vary in more than one dimension (e.g. cost, motivation and resource type: [19]) or require processing of a large amount of information (e.g. numerous options available: [20]). When options are finely balanced (e.g. the net benefit of accessing a large quantity of food with an associated cost is similar to accessing a small quantity of food with no associated cost), individuals are likely to become indifferent to the outcome, choosing both options at approximately equal frequency. When individuals are close to indifference, decisions may not be solved by simple prioritisation (i.e. choosing the alternative with the highest probability-weighted utility) e.g. [21], [22]. Significantly more cognitive effort is therefore invested in finely balanced problems [23] and such decision-making can result in behavioural indicators of frustration and anxiety [24]. In the present study we focussed on comparing the physiology and behaviour of chickens making decisions in which options were either finely balanced or unbalanced.

An unbalanced decision was one in which two options were of unequal net value (i.e. a small quantity of food vs. a large quantity of food). The first part of our study used a titration methodology, in which weighted push-doors were used, to obtain individually-determined points of indifference (i.e. a finely balanced decision). The second part of our study compared the behaviour (head movements and latency to choose) and physiology (surface body temperature and heart-rate variability (HRV)) of individual chickens when making finely balanced and unbalanced decisions. Head movement increases and surface body temperature decreases have been measured in chickens in response to both aversive stimuli (head movements: [25]; surface body temperature: [26]–[28]) and to a signalled palatable reward (head movements: [8], [29]; surface body temperature: [30]). Decreases in HRV, which provides more information on the source of cardiac stimulation (i.e. parasympathetic or sympathetic) than HR [31], occur in response to acutely stressful situations in birds [32]–[34].

We predicted that around the time of finely balanced decision-making, chickens would be more stressed, hence their HRV and surface body temperature would decrease (from levels at the start of the test) compared with an unbalanced alternative. We also predicted that hens would show increased arousal during decision-making (make more head movements) and have increased latencies to choose, due to increased cognitive demand when decisions were finely balanced.

## Methods

### Ethics Statement

All work was conducted under UK Home Office licence (30/2779) and had University ethical approval. We also conducted the study in compliance with ASAB ethical guidelines. The hens were rehomed to small responsible free-range holdings after the study.

### Animals, Housing and Husbandry

Sixteen Columbian Black Tail laying-hens were obtained at approximately 16–18 weeks old from a commercial pullet rearer in Devon. They were transported in poultry crates in a well-ventilated van for approximately 2 h prior to delivery. On arrival all birds were weighed, health-checked, treated with preventative red mite powder (diatomaceous earth: Oak Tree Poultry) and individually tagged for identification using numbered leg rings. They were housed in groups of four, in four out of eight available pens (0.96×1.2 m, 2 m high) in the same room (home room). During weekly cleaning, each group of four birds was switched to the opposite pen within the same room to avoid a housing side-bias. All birds were checked at least once per day and were weighed weekly.

Hens in all pens were fed *ad libitum* feed (Farmgate Layers Mash, BOCM Pauls, Ipswich, Suffolk, UK) via two external feed troughs (total length: 0.77 m), reached by an opening in the pen 0.15 m from floor level. Water was provided via a hanging drinker which was placed in the back corner of each pen (0.2 m high). A nest box (0.39×0.38×0.47 m) and a round perch (0.25 m high, stretching the width of the pen) were also provided. Wood shavings were used as bedding at a depth of 5–10 cm. The room temperature was kept at 19–22°C and the lighting schedule was 12 L: 12 D (light period 7 am–7 pm).

### Experimental room and Procedure

The experimental room contained two pens (one on each side of the room) of the same size as the home room, but which contained no feeders, nest boxes or perches. The experimental pens could be joined by a Perspex tunnel (1.79×0.24 m, 0.47 m high), which formed part of a T-maze apparatus. The experimental room was separated from the home room by solid wooden doors and a corridor, providing an area where hens could be tested away from the noise of conspecifics. Within the experimental room, a CCTV camera was attached to the ceiling above the test apparatus, which was connected to a computer on one side of the room. Another computer for electrocardiogram (ECG) monitoring was set-up on the other side of the room.

### Aim 1 – Establishing Finely Balanced and Unbalanced Decisions

**Figure 1:**
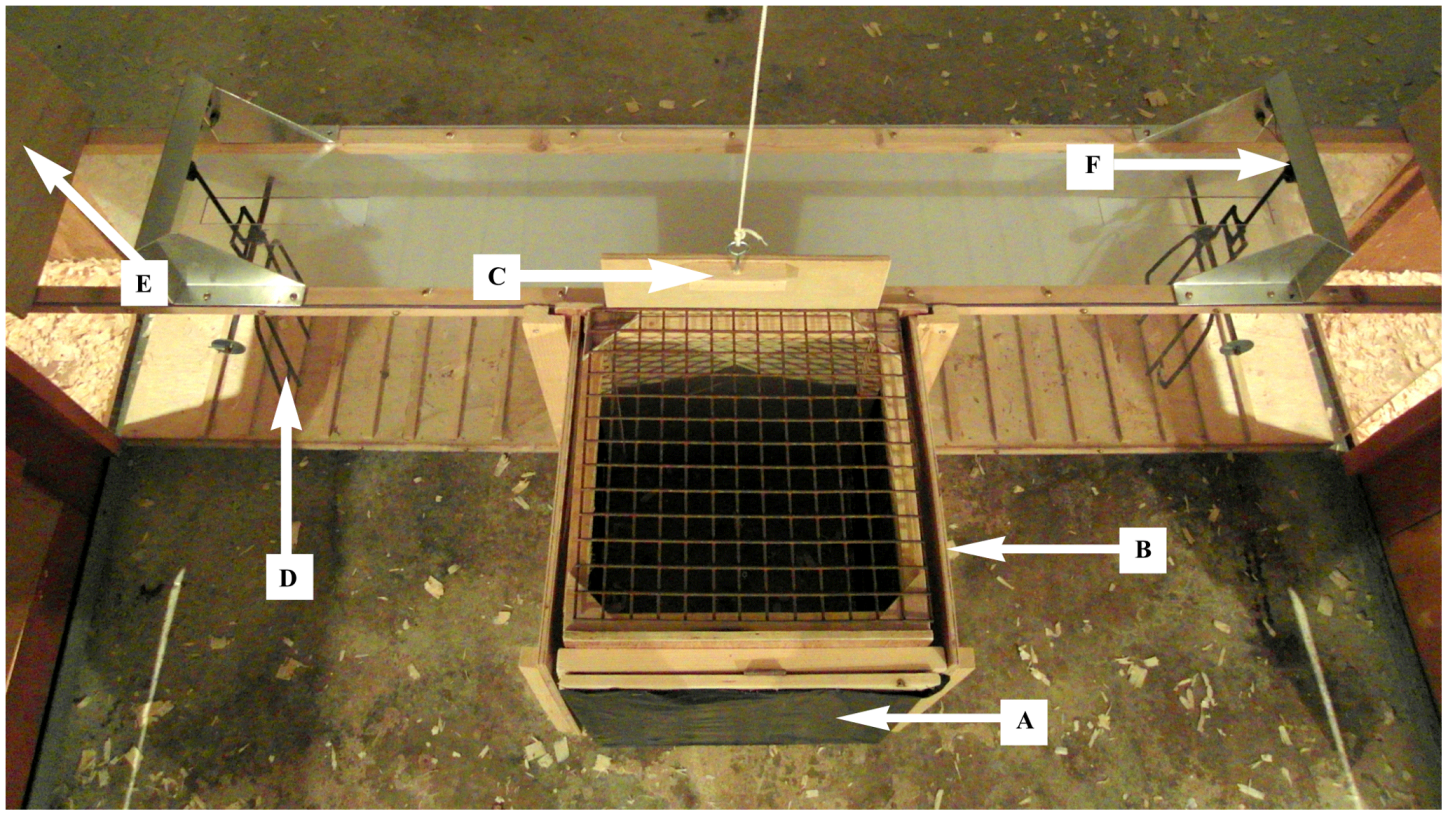
T-maze test apparatus consisting of a Perspex tunnel with push-doors and an attached wooden start-box. The tunnel connects the two pens in the experimental room. **A** indicates the rear of the start-box which was made of black plastic. **B** indicates the wooden side-doors which were removed to reveal wire mesh, through which the feed bowls could be viewed. **C** indicates the tunnel-door, which was raised using a pulley mechanism to allow access to the tunnel. **D** marks the push-doors which were located at either end of the tunnel. **E** marks the pen-door which was placed in the pen entrance once the hen entered the pen, to prevent her from re-entering the tunnel. **F** marks the electromagnet which could be altered to change the force required to open the push-door.

The first aim of this study was to devise a protocol for identifying finely balanced and unbalanced decisions in individual hens. This was achieved using the T-maze test apparatus described above, with incorporated push-doors at each end of the Perspex tunnel. Each push-door could be attached to an electromagnet to alter the force required to push through the door (figure 1). The first step to achieving this aim was to habituate and train the hens. This was followed by a titration procedure.

After a five day settling period, habituation to the ECG monitor, training to establish associations between feed bowls and reward quantity, and habituation to the T-maze test procedure (including push-doors) began. Habituation and training criteria needed to be satisfied at each stage before individuals could progress. Habituation and training took 4–5 weeks, depending on individual progression.

#### ECG recording habituation

We made sure birds were familiar with wearing a harness and an ECG monitor for later phases of the study. ECG was recorded as in [8] using non-invasive remote telemetric units [35] and ECG cables contained within a harness. Hens were gradually habituated to wearing the harness, initially using a simplified harness (with no monitor) for a short period of time. As habituation criteria were met (that hens were able to walk and behave normally in their home environment without moving backwards or stopping excessively), the amount of time wearing the harness was gradually increased in 15 min increments, to a maximum time of 6 h. The ECG cables were then added to the harness and finally the monitor (weighing approx. 100 g). Harness habituation was conducted in both the home and experimental rooms, including within the T-maze.

#### Training associations between feed bowl characteristics and food reward

The hens were individually trained to discriminate between two stimuli (feed bowls), each associated with a different quantity of food (either 1 (Q1) or 6 (Q6) pieces of sweetcorn). Sweetcorn was used instead of a more motivating stimulus like mealworms [36] because we previously found that HR increased considerably in anticipation of a mealworm reward [8] and we wanted to focus on arousal caused by decision-making. During preliminary studies, hens were motivated to eat the sweetcorn reward. We chose to manipulate food quantity rather than quality as we wanted to avoid individual differences in preference for different food types.

**Figure 2:**
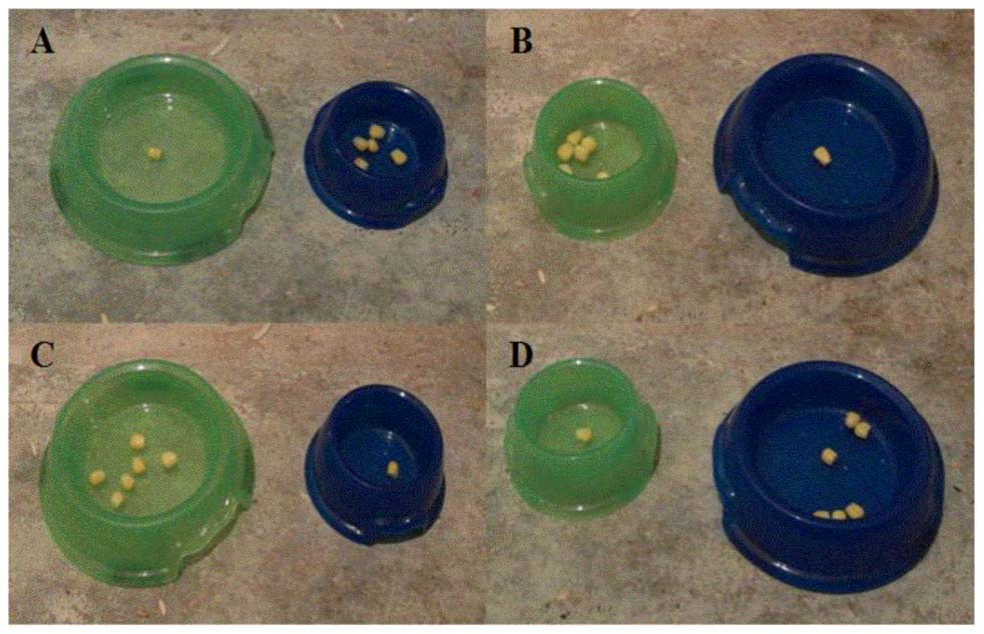
Different coloured and sized feed bowls were used to aid discrimination learning. The feed bowls used were counter-balanced for colour and size, with four chickens being trained on each of the four possible combinations (**A–D**).

The two feed bowls used for each hen were of different size (90 or 140 mm diameter) and colour (green or blue) to aid discrimination of the reward quantity (Q1 and Q6). There were four possible combinations of how size and colour were allocated to reward quantity (figure 2), and bowl combinations were systematically allocated so that one hen from each pen was trained to each. Once hens approached both bowls without stopping or hesitating, discrimination training continued in the T-maze along with habituation to other aspects of the apparatus.

#### T-Maze Habituation

Initial habituation to the T-maze apparatus was conducted in home groups by connecting opposite pens in the home room with a tunnel. Three 3-h group sessions were sufficient to ensure all hens in each group had walked through the tunnel without showing fearful behaviour (stopping or hesitating whilst walking). Habituation then continued individually in the experimental room until hens became accustomed to leaving the start-box and walking through the tunnel with the push-doors (figure 1) fixed open (approximately seven sessions). A tunnel-door suspended by a pulley mechanism was then added to the start-box, to prevent hens from entering the tunnel for a short period at the start of each test (to allow for an initial assessment of behaviour and physiology during the testing phase). To begin with, hens were confined within the start-box for 5 s, but this was gradually increased (when they showed no escape attempts or excessive vocalisations) to 30 s (this took approximately five sessions).

Once accustomed to an initial confinement period, habituation to side-door removal began. The removable wooden side-doors of the start-box were kept in place at the start of each test to prevent hens from viewing the conditioned stimuli (feed bowls placed at both pen entrances). After a 10 s period within the start-box, side-doors were sequentially removed (to ensure that hens were looking at the food bowls on either side of the T-maze) and replaced, then both were removed simultaneously (viewing period). The tunnel-door was then raised and birds were allowed 10 s to leave the start-box (this took approximately six sessions).

When hens had successfully reached criteria in all other aspects of the T-maze procedure, the push-doors (with no weight applied) were introduced. It took approximately eight sessions to train all hens (using sweetcorn) to push through the doors to access the feed bowls. During habituation, a mixture of unidirectional and free-choice trials was conducted and both side-door removal order and the location of each reward quantity (Q1 or Q6) relative to the start-box were systematically varied to ensure equally balanced training. By the end of the training period, hens were well habituated to handling and to all other aspects of the T-maze (including the start-box), having completed approximately 50 individual training trials. If side biases became evident during habituation, additional training was conducted.

Prior to discrimination training in the T-maze, hens were food deprived for an individually-determined period of time to standardise hunger motivation. We selected the minimum food deprivation period (tested during the training phase) that ensured each hen would perform multiple (up to 8) consecutive tests. The required period of food deprivation varied between hens, ranging from 80 to 390 min. As we were using a within-subjects design, the inter-hen range of food deprivation times should not influence treatment-based findings.

When the doors were unweighted and hens consistently chose the Q6 option in the T-maze (at least 90% of the time over consecutive 12 sessions), they were deemed able to discriminate between the two stimuli and this was defined as an unbalanced decision. The mean percentage of Q6 choices made across the 12 consecutive sessions was 97%.

#### Titration Phase

**Figure 3:**
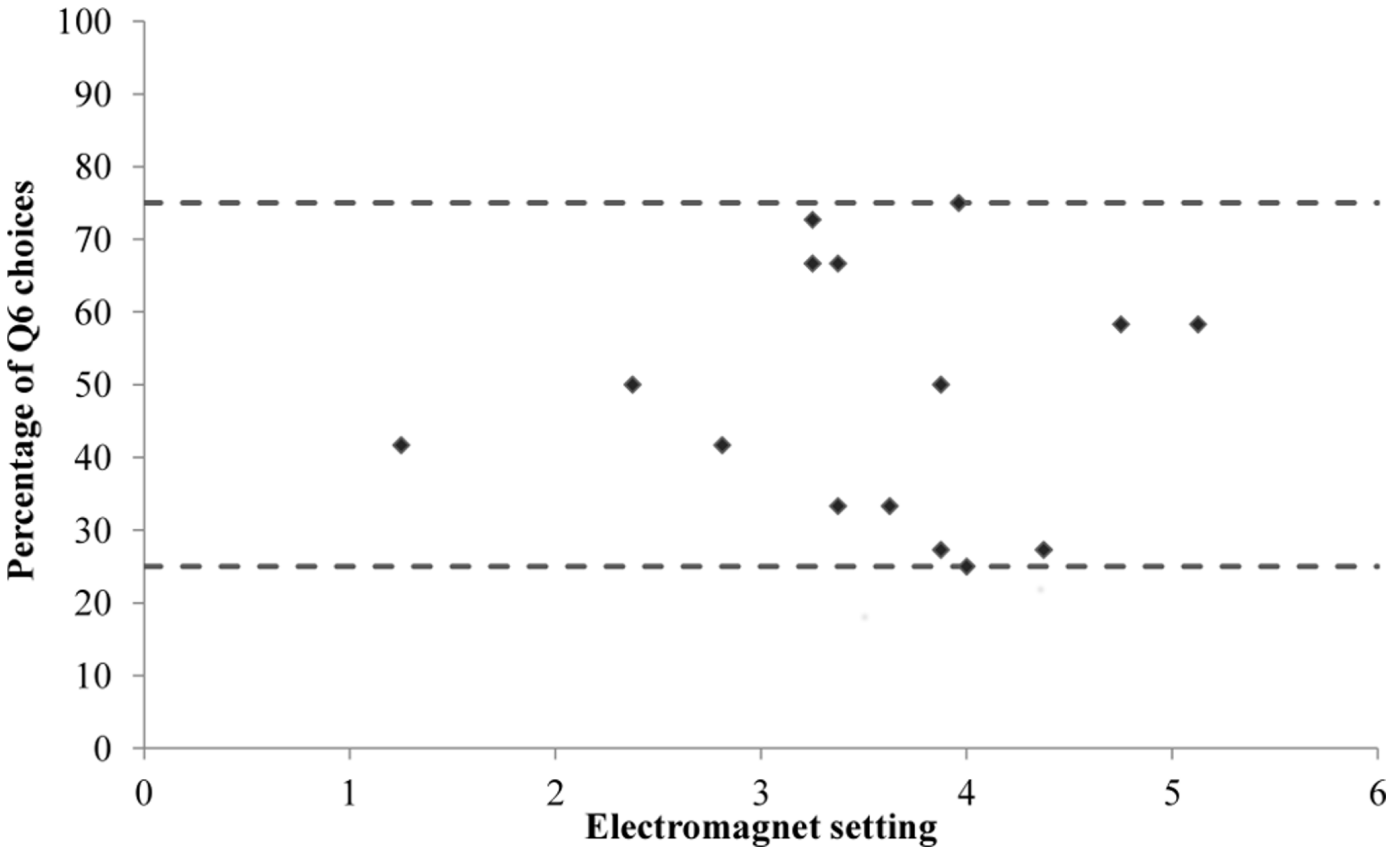
The electromagnet setting at which individual hens chose Q6 within our defined threshold of 25–75% (dashed line) of choices across 12 consecutive trials, measured at the end of the titration phase.

**Figure 4:**
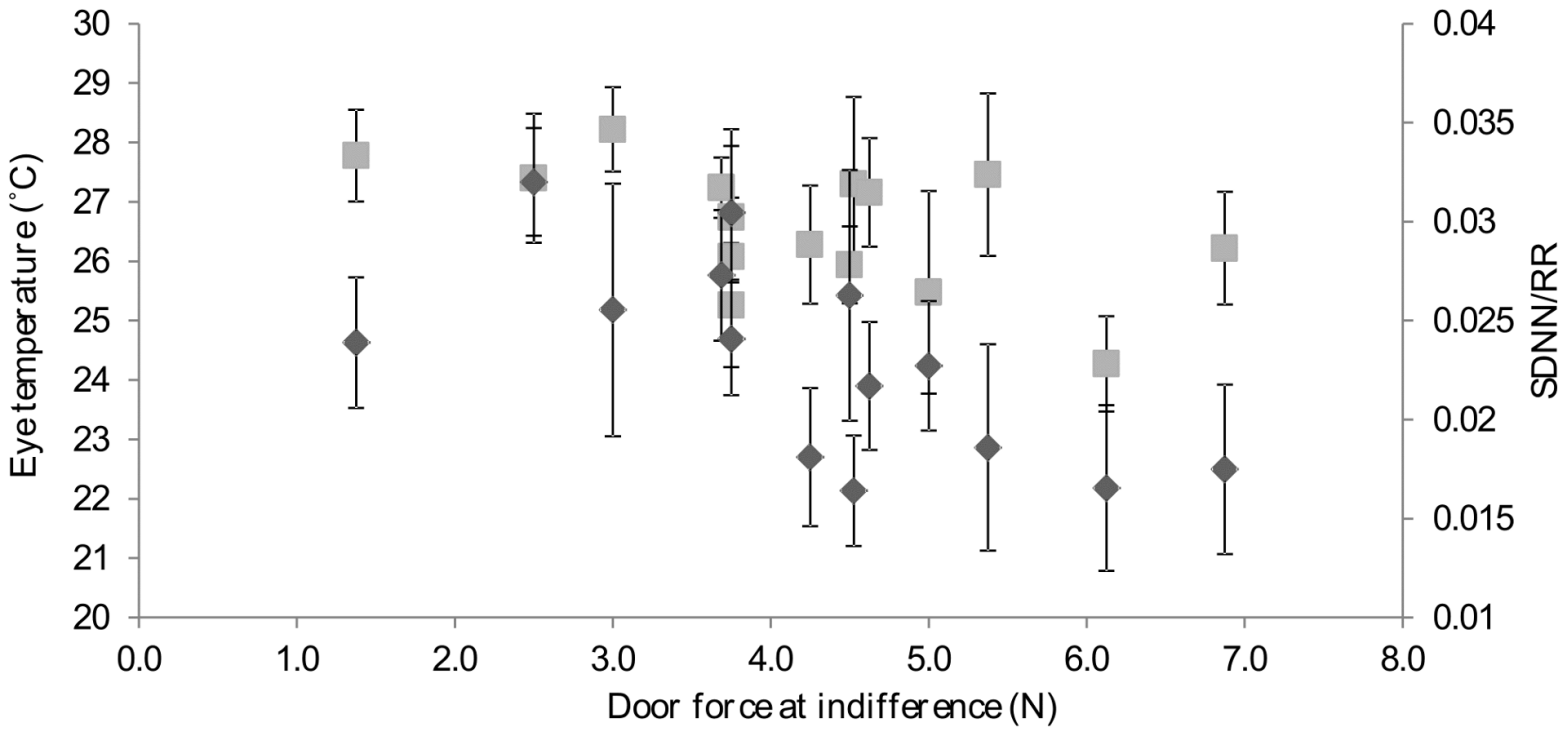
The association between the door force (in Newtons) at indifference and mean ±1 SE SDNN/RR (dark grey) and eye temperature (light grey) during the viewing period when making finely balanced decisions.

The aim of the titration phase was to detect the point at which the two options (Q1 and Q6) had the same net value (i.e. a finely balanced decision). This was done by gradually increasing the force required for hens to push through the Q6 push-door (Q1 door force was always zero), to find the point at which Q6 and Q1 were selected with approximately equal frequency (25–75% of choices) across 12 consecutive sessions. One hen's balance point fell outside the acceptable threshold and the hen was excluded from further testing. A finely balanced decision was defined for the remaining 15 hens – the mean percentage of Q6 choices made across the 12 consecutive sessions was 49% – with the electromagnetic force that had to be applied to the Q6 door varying greatly between individuals (figure 3). The door force at the point of indifference can be seen in figure 4.

### Aim 2 – Behavioural and Physiological Responses to Balanced and Unbalanced Decisions

**Figure 5:**
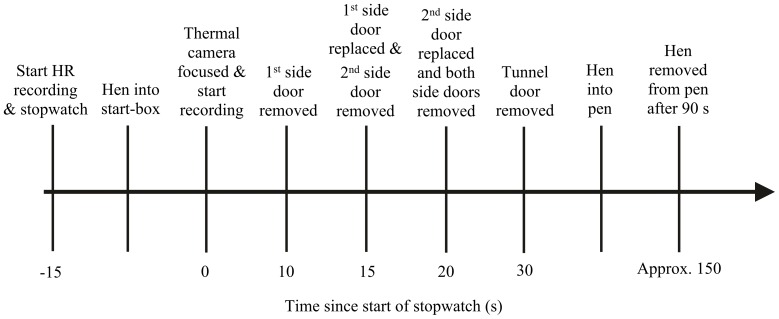
The test procedure during the testing phase. A stopwatch was used to monitor the precise time at which each event occurred so that accurate start-box and viewing period HRV and temperatures could be extracted from the data files.

Once the unbalanced and finely balanced decisions had been individually defined, each hen underwent 10 consecutive days of testing during which aspects of their behaviour and physiology were monitored. On each day (which involved either an unbalanced or a finely balanced decision), hens were food deprived as for training, before being given two unidirectional (forced) trials (one to either option) followed by one free-choice test (when the door weights remained as in the forced trials). The forced trials allowed hens to ‘assess’ whether the free-choice test would be unbalanced or finely balanced. The test protocol is outlined in figure 5. Prior to starting each free-choice test, the ECG monitor and a stopwatch were activated simultaneously. The test commenced when hens were placed in the start-box and confined for 10 s with the wooden side-doors in place, so that initial (start-box) physiological measures could be taken without conditioned stimuli (feed bowls) being visible. The side-doors were sequentially removed from the start-box for 5 s and replaced, and then both side-doors were simultaneously removed for a 10 s viewing period. The tunnel-door was then raised, allowing access to the push-doors (‘the push-door period’). Once hens had accessed their chosen feed bowl via the push-door, the pen-door was closed and they were confined within the pen for 90 s (to allow consumption of their reward: ‘the pen period’). If a hen failed to enter the tunnel within the first 60 s of each test, she was gently encouraged into the tunnel and the tunnel-door was replaced. A maximum time of 300 s was given for hens to enter a pen, after which time the test was stopped and the hen was removed from the T-maze.

Only one free-choice test was given each day to ensure that hunger motivation was kept as constant as possible throughout the experiment. Tests were carried out on each individual at the same time of day across the testing phase. Five tests were conducted per hen for both finely balanced and unbalanced decisions during the testing period. The order in which each hen experienced each decision category was systematically alternated, with no more than two consecutive tests from either decision category. The side of the T-maze where Q1 and Q6 were presented, the order of unidirectional trials and the order of side-door removal were also systematically alternated using the same criteria. Side biases were investigated and checked as the experiment progressed. The unidirectional trials given ensured that hens visited both sides of the T-Maze.

For each free-choice test, the following measures were taken during the push-door period: the latency to the push-door, the number of switches between push-doors and the number of attempts made to enter the pen. The side of the T-maze which hens first tried to enter was recorded as their ‘first push’ and their ‘ultimate choice’ was also noted. The latency to the push-door and latency to the pen were both recorded as outlined in figure 5. A CCTV camera was fixed above the start-box and video was continuously recorded using WebCCTV software. The videos were analysed using The Observer Software version 10.0 to measure the number of head movements during the 10 s viewing period.

Throughout each free-choice test, the ECG was recorded continuously onto a micro-SD flash card which was inserted into the monitor. The monitor communicated with a base unit (attached to a computer via USB connection) and was controlled using RVC Telemetry Software version 1.5. HRV data were extracted using Spike 2 Software (version 6) from three 10 s periods: start-box, viewing and pen. As we were primarily interested in whether more difficult decisions induced arousal, we extracted two measures of HRV which are influenced by both branches of the autonomic nervous system [37] – mean interbeat interval (RR) and the quotient of the standard deviation of interbeat intervals and RR (i.e. the coefficient of variance (SDNN/RR)). Measures of HRV were taken at the start of each test (start-box period) to check for individual differences and to account for the influence of handling on arousal.

Surface body temperature was also recorded during the start-box and viewing periods using a thermal video camera (FLIR SC305), and was extracted using FLIR ResearchIR Software version 1.2 SP2. The rear surface of the wooden start-box was made from black plastic (through which we were able to detect heat radiation from the chicken), allowing us to record surface body temperature without hens being able to view the experimenter. The emissivity of the black plastic was calculated to be 0.292. A clear image of the side of the head was taken from each video to obtain the eye and maximum head temperature (hottest part of exposed skin). Video was recorded at 3 frames/second so it was usually possible to obtain clear images even if hens were moving.

### Statistical Analysis

The data were analysed using *IBM SPSS Statistics 19*. Fifteen hens were given 10 free-choice tests each. HRV data were not collected from one individual as she did not behave normally when wearing the monitor during training. This hen did not wear the monitor during testing, hence we did not collect HRV data from her reducing the HRV sample to 14 individuals. Temperature, head movement and latency data were collected for all 15 individuals. Prior to conducting analyses we investigated the possibility of interactions by visual, inspection of plotted results for all measured variables and found no evidence of such interactions. For each set of data, the assumptions of parametric testing were checked and data were transformed if possible, then analysed using paired-samples t-tests. Where transformations were not possible or unsatisfactory, Wilcoxon tests were used. For analyses, a mean of each measure (% Q6 choices, RR intervals, SDNN/RR, head movements, maximum head and eye temperature, latency to first push and pen, number of switches made, number of attempts to enter the pen) was taken from the five repeats to each hen, to look for differences between finely balanced and unbalanced decisions during the relevant periods (start-box, viewing, push-door, pen). Unless otherwise stated, means ± SE are presented (in figures or text) for parametric data and the median, interquartile range (IQR) and range are presented for non-parametric data as appropriate. A measure of effect size is given alongside significant results. As means were used for the analyses, the range of individual hen standard deviations is given where appropriate.

## Results

### Aim 1 – Establishing Finely-Balanced and Unbalanced Decisions

To validate the titration procedure, the percentage of Q6 choices made during the testing phase was calculated. Overall, significantly more Q6 choices were made in the unbalanced condition (median: 100; IQR: 60–100; range: 60–100%) than in the finely balanced condition (median: 60; IQR: 40–80; range: 0–100%; Wilcoxon signed rank test: *z* = 2.83, *n* = 15, *p*<0.01, *r* = 0.52).

### Aim 2 – Behavioural and Physiological Responses to Balanced and Unbalanced Decisions

#### Start-box period

**Figure 6:**
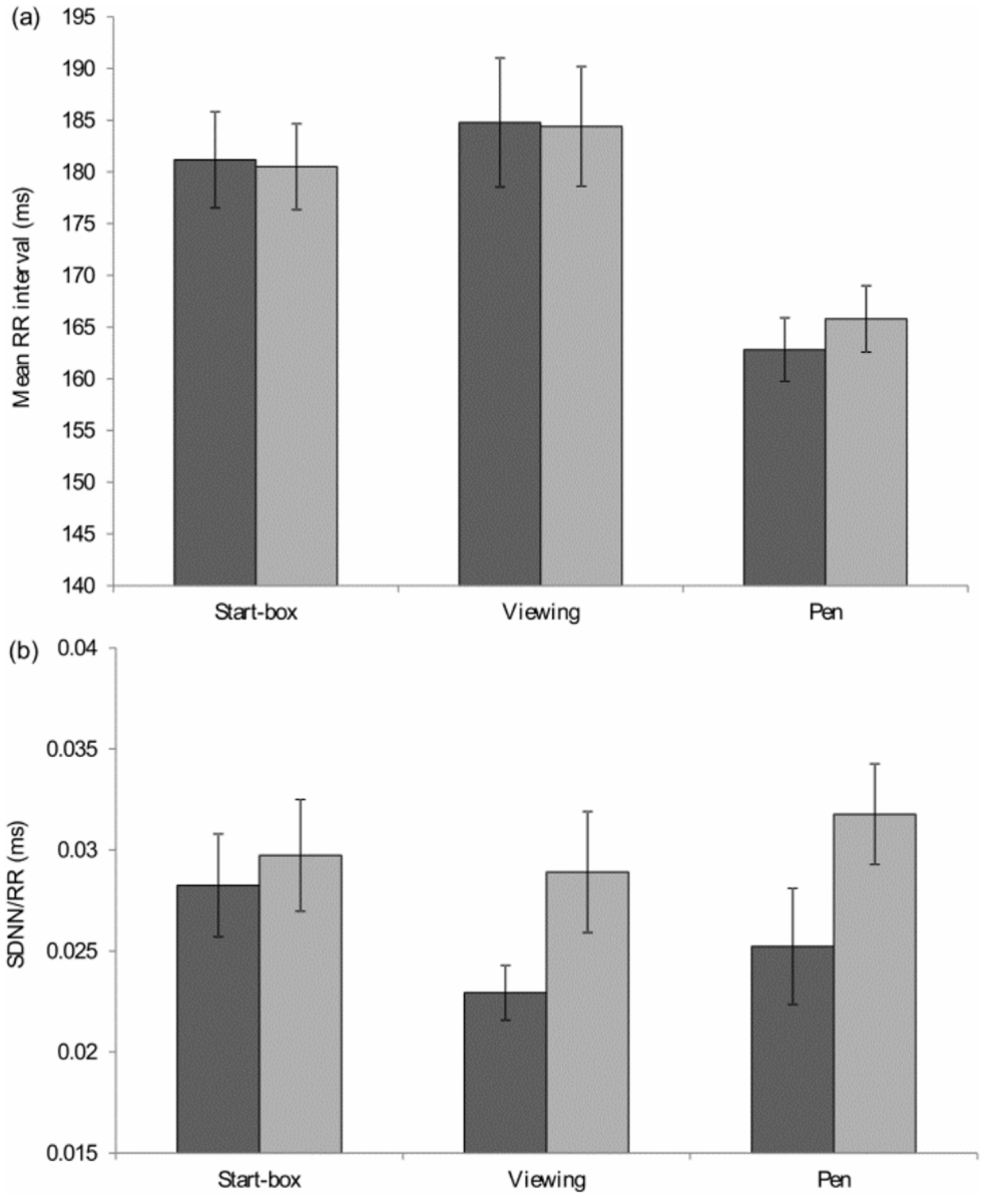
Mean ±1 SE (a) RR interval, (b) coefficient of variance (SDNN/RR), during each period within the test when making finely balanced (dark grey) and unbalanced (light grey) decisions.

**Figure 7:**
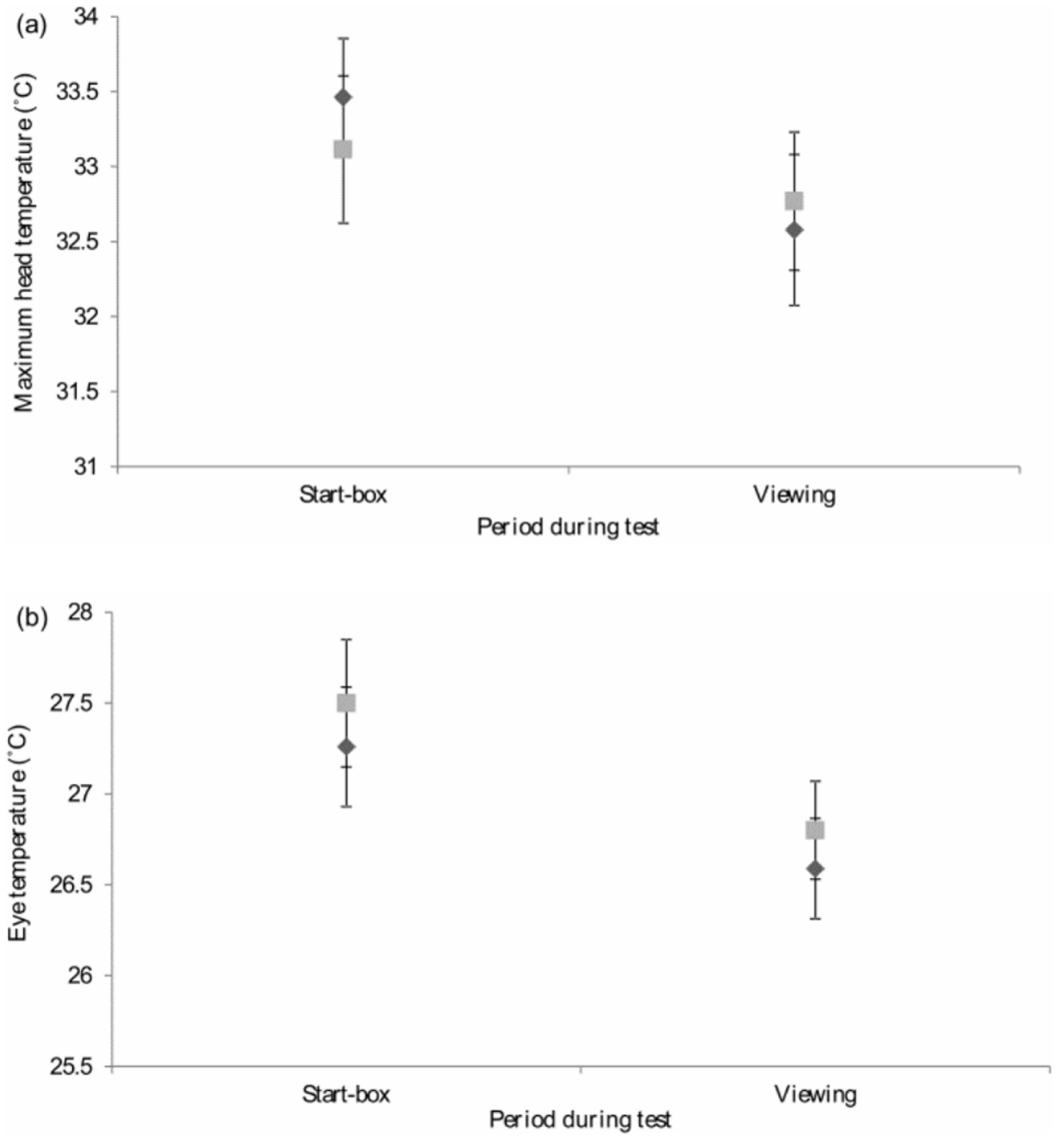
Mean ±1 SE (a) maximum head temperature, (b) eye temperature, during the start-box and viewing periods when making finely balanced (dark grey) and unbalanced (light grey) decisions.

There were no significant differences between treatments in RR intervals (range of individual standard deviation: finely balanced  = 3.5–22.4 ms, unbalanced  = 4.3–10.7 ms; paired samples t-test: *t*_13_  = 0.43, *p* = 0.68, figure 6*a*) or in SDNN/RR (range of individual standard deviation: finely balanced  = 0.004–0.03, unbalanced  = 0.004–0.03; paired samples t-test: *t*_13_  = 0.72, *p* = 0.49, figure 6*b*). There were also no significant differences in start-box maximum head temperature (range of individual standard deviation: finely balanced  = 0.9–5.2°C, unbalanced  = 1.2–4.1°C; paired samples t-test: *t*_14_  = 0.91, *p* = 0.38, figure 7*a*) or eye temperature (range of individual standard deviation: finely balanced  = 1.3–3.8°C, unbalanced  = 1.4–4.2°C; paired samples t-test: *t*_14_  = 0.71, *p* = 0.49, figure 7*b*) between finely balanced and unbalanced decisions.

#### Viewing period

During the viewing period, there was no significant difference in RR between finely balanced and unbalanced decisions (range of individual standard deviation: finely balanced  = 2.4–15.0 ms, unbalanced  = 2.7–13.8 ms; paired samples t-test: *t*_13_  = 0.28, *p* = 0.79, figure 6*a*). However, there was a strong trend for a lower SDNN/RR when making a finely balanced decision compared to an unbalanced one (range of individual standard deviation: finely balanced  = 0.006–0.01, unbalanced  = 0.002–0.05; *t*_13_  = 2.10, *p* = 0.055, figure 6*b*). There was a significant negative correlation between the door weight at indifference and SDNN/RR during the viewing period (Pearson correlation coefficient  = −0.68, *n* = 14, *p* = 0.008, figure 4). There were no significant differences between treatments in the number of head movements (range of individual standard deviation: finely balanced  = 0.7–3.8, unbalanced  = 0.5–2.9; *t*_14_  = 0.26, *p* = 0.80) or in the maximum head (range of individual standard deviation: finely balanced  = 1.3–4.2°C, unbalanced  = 1.2–3.7°C; *t*_14_  = 0.65, *p* = 0.52, figure 7*a*) and eye temperatures (range of individual standard deviation: finely balanced  = 1.1–3.8°C, unbalanced  = 1.5–3.7°C; *t*_14_  = 0.81, *p* = 0.43, figure 7*b*). There was also a significant negative correlation between the door weight at indifference and the eye temperature during the viewing period (Pearson correlation coefficient  = 0.55, *n* = 15, *p* = 0.033, figure 4).

#### Push-door period

**Figure 8:**
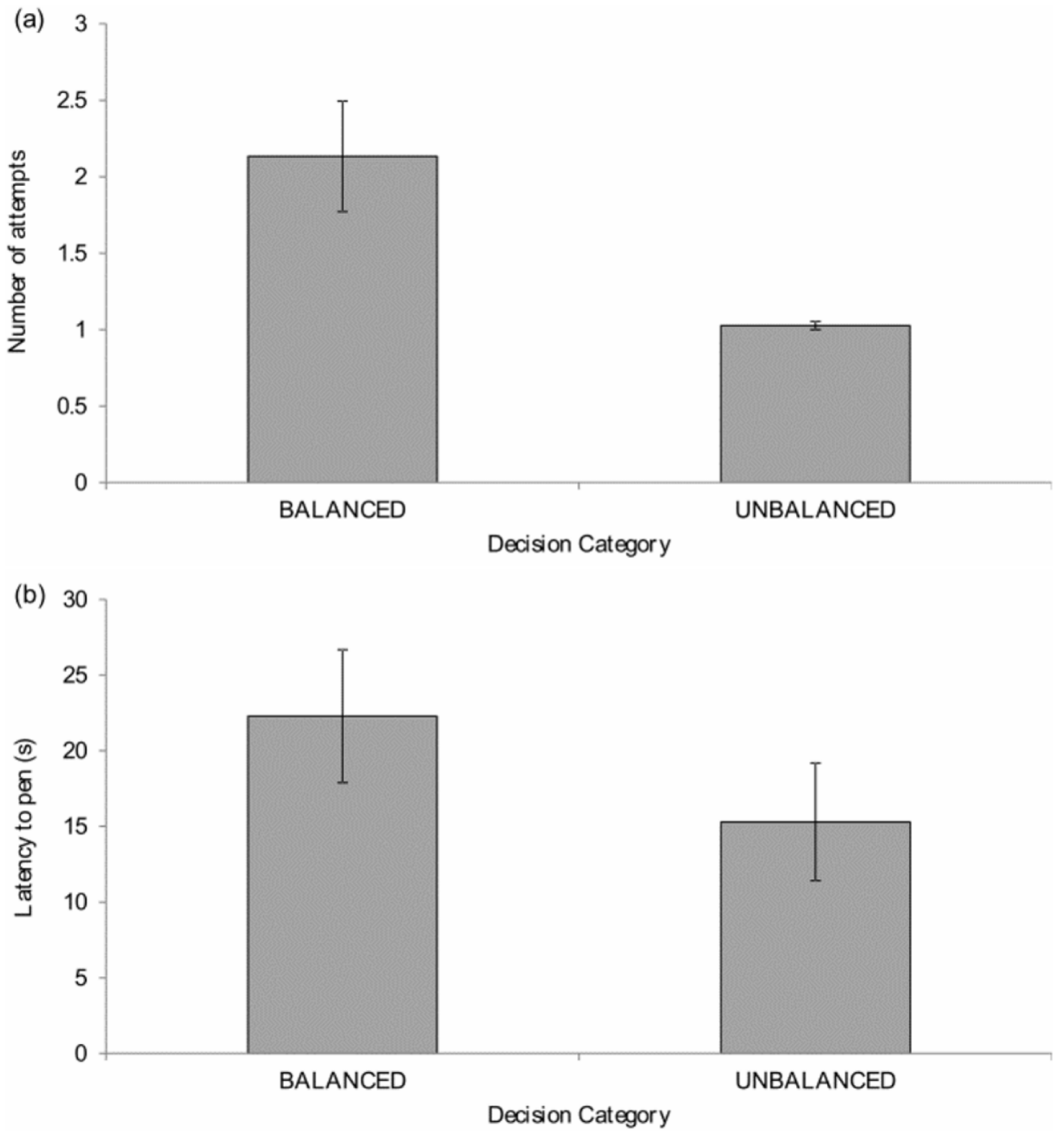
Mean ±1 SE (a) number of attempts made, (b) latency to pen.

Latency to the first push on a door was not significantly influenced by experimental condition (range of individual standard deviation: finely balanced  = 1.0–36.5 s, unbalanced  = 0.4–50.1 s; paired-samples t-test: *t*_14_  = 0.08, *p* = 0.94). Hens did make significantly more switches between push-doors (finely balanced: median: 0.2; range: 0–0.6; unbalanced: median: 0; range: 0–0; Wilcoxon signed rank test: *z* = 2.59, *n* = 15, *p* = 0.01, *r* = 0.47) and significantly more attempts to enter the pen (finely balanced: median: 1.6; range: 1–6.2; unbalanced: median: 1; range: 1–1.4; *z* = 3.06, *n* = 15, *p*<0.01, *r* = 0.56, figure 8*a*) in the finely balanced treatment compared to when the decision was unbalanced.

#### Pen period

Hens took significantly longer to reach the pen when making finely balanced decisions compared to unbalanced ones (range of individual standard deviation: finely balanced 1.6–56.5 s, unbalanced  = 0.5–68.5 s; paired samples t-test: *t*_14_  = 2.73, *p* = 0.02, eta squared  = 0.35, figure 8*b*). Once in the pen, the RR interval was significantly shorter (range of individual standard deviation: finely balanced  = 3.0–20.8 ms, unbalanced  = 1.9–9.9 ms; *t*_13_  = 3.22, *p* = 0.007, eta squared  = 0.46, figure 6*a*) and the SDNN/RR was significantly lower (range of individual standard deviation: finely balanced  = 0.001–0.05, unbalanced  = 0.005–0.03; *t*_13_  = 2.46, *p* = 0.029, eta squared  = 0.34, figure 6*b*) when hens had made finely balanced decisions compared to unbalanced ones.

## Discussion

Our titration methodology, based on the idea that two options are substitutable when they are of equal net value [16], was successful in determining the cost (the force needed to open the access door) required for hens to choose a larger food reward at an equivalent frequency to a smaller food reward. The electromagnetic force required to generate finely balanced choices varied between individuals, highlighting the importance of conducting this protocol on an individual hen basis. Finely balanced decisions between two substitutable options are likely to be inherently more difficult than unbalanced decisions [16], [23], [24], so our method allowed subsequent testing of behavioural and physiological correlates of this aspect of decision difficulty in hens. The titration method we have developed could also prove useful in examining the effect of this aspect of decision difficulty in other species.

Three significant physiological and behavioural differences were found between the finely balanced and unbalanced conditions, but all of these occurred outside the ‘viewing period’ (when we expected hens to make their decision and that arousal caused by decision-making would be detectable) after hens had left the start-box. Measures were taken during the push-door and pen periods to monitor the effect of the decision consequence on arousal. Under finely balanced conditions, hens took longer to reach the pen and there were differences in the two measures of HRV: mean RR interval was significantly shorter and the coefficient of variance (SDNN/RR) was significantly lower when hens had made finely balanced decisions. These differences are likely to be artefacts of the additional time and effort caused by hens having to push against the weighted door, of them switching more often between push-doors, and of additional attempts to enter the pen in the finely balanced condition. We had predicted that decreases in measures of HRV in the pen and longer latencies to pen in the finely balanced condition would be accompanied by a decrease in HRV during the viewing period and an increased latency to the first push, if affected by the difficulty of the decision. Although the coefficient of variance was influenced by experimental condition, the difference was not quite statistically significant, and there were no treatment-related differences in latency to the first push and the RR interval, suggesting that the observed differences in pen measures were unlikely to be caused by the difficulty of decision-making. We did find however, that the door weight at indifference was negatively correlated with some measures of arousal during the viewing period (eye temperature and the coefficient of variance), suggesting that hens pushing heavier doors may have anticipated the extra weight.

We found no other significant influences of experimental condition on the eye and maximum head temperature change or on the number of head movements made during decision-making. This could be interpreted in several ways. First, although the perceived difficulty of a decision affects human behaviour and physiology [4]–[7], it might not result in the same responses around the time of decision-making in other animals or more specifically in birds. However, previous research has shown that some non-human animals, such as dolphins, gorillas and honey bees, behave similarly to humans during other types of difficult tasks [18], [38], [39]. The key difference may therefore relate to the manner in which a decision is defined as difficult. In the aforementioned studies, the type of difficult decisions examined tends to involve a degree of uncertainty, for example as a result of ambiguous cues. And in the studies of human arousal during decision-making, difficult decisions often involved a degree of risk [5], [6], [7]. These are factors that we are currently examining in separate work, but here we explored behavioural and physiological effects of an aspect of decision difficulty that has not previously been examined in other species (i.e. a decision between two options of equal net value).

One reason why the birds were not aroused when making finely balanced decisions may have been because they adopted a simple non-arousing strategy to deal with the situation. When humans must make decisions but are indifferent to the outcomes, they can adopt strategies such as choosing randomly, consistently choosing one option (status quo maintenance) or alternating between options e.g. [40]–[42]. Although potentially costly in terms of time [23], the use of non-evaluative decision strategies may be less arousing than evaluative decision-making in humans, though to our knowledge this has not been tested. Possibly our finely balanced decisions were difficult, but not stressful enough to result in physiological arousal.

One additional possibility is that the overall method and the behavioural and physiological measures we took were not sensitive enough to detect changes during the relevant period. However, we have previously found strongly significant changes in these measures in our related studies with chickens [8] and others have found differences in these measures in anticipation of and in response to positive and negative stimuli [25], [27], [29], [30]. Often during human decision-making experiments, antecedents and consequences of decision-making are monitored, rather than directly measuring changes during the process, due to the difficulty of identifying the exact point at which decisions are made. For the purpose of our current experiment, we considered that the birds' decision period most likely coincided with the 10-s viewing period, when the hens could view options but not yet access them. It is possible that hens did not make their choice during our defined decision period, or that changes in arousal occurred later than we had predicted. The coefficient of variance (HRV) showed a strong trend (*p* = 0.055) towards a significant difference between our experimental conditions which suggests that some decreases in HRV occurred during the viewing period. It is possible that a time-lag between decision-making and the onset of physiological arousal exists.

Although the titration methodology we developed was successful in identifying finely balanced and unbalanced decisions in individual chickens, to progress work in this area more information is needed on when best to monitor changes in arousal during decision-making. Additionally, future work could aim to identify whether such finely balanced decisions result in behavioural and physiological arousal in other species, and to explore alternative definitions of difficult decisions. As experimental protocols involving decision-making are used widely in animal welfare research [9]–[11], it is essential that all procedural influences are taken into consideration for an accurate interpretation of results.

## References

[1] Saaty (1988) What is the analytic hierarchy process? In: Mitra G, Greenberg HJ, Lootsma FA, Rijckaert MJ, Zimmerman HJ, editors. Mathematical models for decision support. NATO ASI Series Vol 48. Springer Berlin Heidelberg. pp.109–121.

[2] McFarland DJ (1977) Decision making in animals. Nature 269: 15–21.

[3] Crone EA, Somsen RJM, Van Beek B, Van Der Molen MW (2004) Heart rate and skin conductance analysis of antecendents and consequences of decision-making. Psychophysiology 41: 531–540. 15189476 10.1111/j.1469-8986.2004.00197.x

[4] Gerard HB (1967) Choice difficulty, dissonance and the decision sequence. J Pers 35: 91–108. 6031221 10.1111/j.1467-6494.1967.tb01417.x

[5] Mann L, Janis IL, Chaplin R (1969) Effects of anticipation on predecisional processes. J Pers Soc Psychol 11: 10–16.

[6] Critchley HD, Mathias J, Dolan RJ (2001) Neural activity in the human brain relating to uncertainty and arousal during anticipation. Neuron 29: 537–545. 11239442 10.1016/s0896-6273(01)00225-2

[7] Van Harreveld F, Rutjens BT, Rotteveel M, Nordgren LF, van der Pligt J (2009) Ambivalence and decisional conflict as a cause of psychological discomfort: feeling tense before jumping off the fence. J Exp Soc Psychol 45: 167–173.

[8] Davies AC, Radford AN, Nicol CJ (2014) Behavioural and physiological expression of arousal during decision-making in laying hens. Physiol Behav 123: 93–99. 24432355 10.1016/j.physbeh.2013.10.008 PMC3866659

[9] Kirkden RD, Pajor EA (2006) Using preference, motivation and aversion tests to ask scientific questions about animals' feelings. Appl Anim Behav Sci 100: 29–47.

[10] Jensen MB, Pedersen LJ (2008) Using motivation tests to assess ethological needs and preferences. Appl Anim Behav Sci 113: 340–356.

[11] Fraser D, Nicol CJ (2011) Preference and motivation research. In: Appleby MC, Mench JA, Olsson IAS, Hughes BO, editors. Animal Welfare 2nd Edition. CABI. pp.183–199.

[12] Shields SJ, Garner JP, Mench JA (2004) Dustbathing by broiler chickens: a comparison of preference for four different substrates. Appl Anim Behav Sci 87: 69–82.

[13] Widowski TM, Keeling LJ, Duncan IJH (1992) The preferences of hens for compact fluorescent over incandescent lighting. Can J Anim Sci 72: 203–211.

[14] Bateson M (2004) Mechanisms of decision-making and the interpretation of choice tests. Anim Welf 13: 115–120.

[15] Browne WJ, Caplen G, Statham P, Nicol CJ (2011) Mild environmental aversion is detected by a discrete-choice preference testing method but not by a free-access method. Appl Anim Behav Sci 134: 152–163.

[16] Sørensen DB, Ladewig J, Ersbøl AK, Matthews L (2004) Using the cross point of demand functions to assess animal priorities. Anim Behav 68: 949–955.

[17] Lima SL, Dill LM (1990) Behavioural decisions made under the risk of predation: a review and prospectus. Can J Zool 68: 619–640.

[18] Perry CJ, Barron AB (2013) Honey bees selectively avoid difficult choices. Proc Natl Acad Sci USA 110: 19155–19159. 24191024 10.1073/pnas.1314571110 PMC3839751

[19] Cooper JJ, Mason GJ (2000) Increasing costs of access to resources cause re-scheduling of behaviour in American mink (*Mustela vison*): implications for the assessment of behavioural priorities. Appl Anim Behav Sci 66: 135–151.

[20] Churchland AK, Kiani R, Shadlen MN (2008) Decision-making with multiple alternatives. Nat Neurosci 11: 693–702. 18488024 10.1038/nn.2123 PMC2453226

[21] Manski CF (1975) Maximum score estimation of the stochastic utility model of choice. J Econom 3: 205–228.

[22] Varian HR (1990) Goodness-of-fit in optimizing models. J Econom 46: 125–140.

[23] Moffatt PG (2005) Stochastic choice and the allocation of cognitive effort. Exp Econ 8: 369–388.

[24] Ventricelli M, Focaroli V, De Petrillo F, Macchitella L, Paglieri F, Addessi E (2013) How capuchin monkeys (Cebus apella) behaviourally cope with increasing delay in a self-control task. Behav Process 100: 146–152. 10.1016/j.beproc.2013.09.001 24056241

[25] Zimmerman PH, Buijs SAF, Bolhuis JE, Keeling LJ (2011) Behaviour of domestic fowl in anticipation of positive and negative stimuli. Anim Behav 81: 569–577.

[26] Cabanac M, Aizawa S (2000) Fever and tachycardia in a bird (*Gallus domesticus*) after simple handling. Physiol Behav 69: 541–545. 10913794 10.1016/s0031-9384(00)00227-4

[27] Edgar JL, Lowe JC, Paul ES, Nicol CJ (2011) Avian maternal response to chick distress. Proc R Soc B 278: 3129–3134. 10.1098/rspb.2010.2701 PMC3158930 21389025

[28] Edgar JL, Nicol CJ, Pugh CA, Paul ES (2013) Surface temperature changes in repsonse to handling in domestic chickens. Physiol Behav 119: 195–200. 23816981 10.1016/j.physbeh.2013.06.020

[29] Moe RO, Nordgreen J, Janczak AM, Spruijt BM, Zanella AJ, et al (2009) Trace classical conditioning as an approach to the study of reward-related behaviour in laying hens: A methodological study. Appl Anim Behav Sci 121: 171–178.

[30] Moe RO, Stubsjøen SM, Bohlin J, Flø A, Bakken M (2012) Peripheral temperature drop in response to anticipation and consumption of a signaled palatable reward in laying hens *(Gallus domesticus)* . Physiol Behav 106: 527–533. 22513240 10.1016/j.physbeh.2012.03.032

[31] Von Borell E, Langbein J, Després G, Hansen S, Leterrier C, et al (2007) Heart rate variability as a measure of autonomic regulation of cardiac activity for assessing stress and welfare in farm animals – A review. Physiol Behav 92: 293–316. 17320122 10.1016/j.physbeh.2007.01.007

[32] Kjaer JB, Jørgensen H (2011) Herat-rate variability in domestic chicken lines genetically selected on feather pecking behaviour. Genes Brain Behav 10: 747–755. 21682845 10.1111/j.1601-183X.2011.00713.x

[33] Cyr NE, Dickens MJ, Romero ML (2009) Heart-rate and heart-rate variabilirty responses to acute and chronic stress in a wild-caught passerine bird. Physiol Biochem Zool 82: 332–344. 19115847 10.1086/589839

[34] Korte SM, Ruesnik W, Blokhuis HJ (1999) Heart-rate variability during manual restraint in chicks from high- and low-feather pecking lines of laying hens. Physiol Behav 65: 649–652. 10073462 10.1016/s0031-9384(98)00206-6

[35] Lowe JC, Abeyesinghe SM, Demmers TGM, Wathes CM, McKeegan DEF (2007) A novel telemetric logging system for recording physiological signals in unrestrained animals. Comput Electron Agr 57: 74–79.

[36] Bruce EH, Prescott NB, Wathes CM (2003) Preferred food rewards for laying hens in behavioural experiments. Br Poult Sci 44: 345–349. 12964615 10.1080/0007166031000085490

[37] Task Force of the European Society of Cardiology, North American Society of Pacing and Electrophysiology (1996) Heart rate variability: standards of measurement, physiological interpretation and clinical use. Circulation 93: 1043–1065. 8598068

[38] Smith DJ, Schull J, Strote J, McGee K, Egnor R, et al (1995) The uncertain response in the bottlenosed dolphin (*Tursiops truncatus*). J Exp Psychol 124: 391–408. 10.1037//0096-3445.124.4.391 8530911

[39] Suda-King C, Bania AE, Stromberg EE, Subiaul F (2013) Gorillas' use of the escape response in object choice memory tests. Animal Cognition 16: 65–84. 22923213 10.1007/s10071-012-0551-5

[40] Mandler M (2005) Incomplete preferences and rational intransitivity of choice. Games Econ Behav 50: 255–277.

[41] Eliaz K, Ok EA (2006) Indifference or indecisiveness? Choice theoretic foundations of incomplete preferences. Games Econ Behav 56: 61–86.

[42] Danan E (2010) Randomization vs. Selection: How to choose in the absence of preference? Manage Sci 56: 503–518.

